# Defining the male contribution to embryo quality and offspring health in assisted reproduction in farm animals

**DOI:** 10.1590/1984-3143-AR2020-0018

**Published:** 2020-08-05

**Authors:** Hannah Louise Morgan, Nader Eid, Afsaneh Khoshkerdar, Adam John Watkins

**Affiliations:** 1 Division of Child Health, Obstetrics and Gynaecology, Queen’s Medical Centre, School of Medicine, University of Nottingham, Nottingham, United Kingdom

**Keywords:** assisted reproductive technologies, fetal programming, semen quality

## Abstract

Assisted reproductive technologies such as artificial insemination have delivered significant benefits for farm animal reproduction. However, as with humans, assisted reproduction in livestock requires the manipulation of the gametes and preimplantation embryo. The significance of this ‘periconception’ period is that it represents the transition from parental genome regulation to that of the newly formed embryo. Environmental perturbations during these early developmental stages can result in persistent changes in embryonic gene expression, fetal organ development and ultimately the long-term health of the offspring. While associations between maternal health and offspring wellbeing are well-defined, the significance of paternal health for the quality of his semen and the post-conception development of his offspring have largely been overlooked. Human and animal model studies have identified sperm epigenetic status (DNA methylation levels, histone modifications and RNA profiles) and seminal plasma-mediated maternal uterine immunological, inflammatory and vascular responses as the two central mechanisms capable of linking paternal health and post-fertilisation development. However, there is a significant knowledge gap about the father’s contribution to the long-term health of his offspring, especially with regard to farm animals. Such insights are essential to ensure the safety of widely used assisted reproductive practices and to gain better understanding of the role of paternal health for the well-being of his offspring. In this article, we will outline the impact of male health on semen quality (both sperm and seminal plasma), reproductive fitness and post-fertilisation offspring development and explore the mechanisms underlying the paternal programming of offspring health in farm animals.

## Introduction

**Figure 1:**
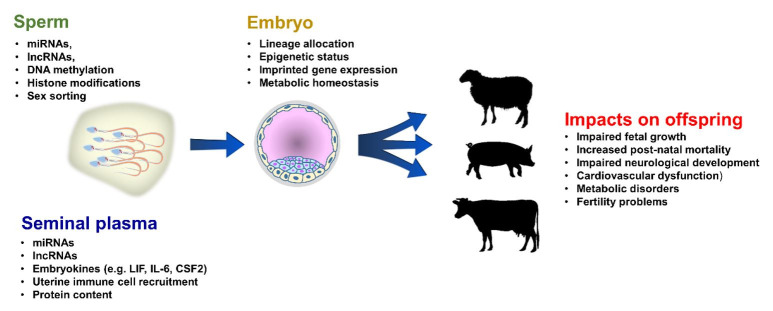
The paternal influence on semen quality, embryo development and offspring health. A range of male factors including nutritional status, age and stress and sperm manipuation can impact on sperm factors including miRNA (microRNA), lncRNA (long-non coding RNA) and DNA integrity. Male health can also affect seminal plasma composition including proteins, cytokines and exosomes. Both sperm and seminal plasma can influence embryo development and long-term offspring development and well-being.

The development of efficient assisted reproductive technologies (ART) such as artificial insemination in cattle has increased the genetic gain in livestock dramatically (Pellegrino et al., 2016). This has resulted in enhanced productivity and health in multiple cattle, swine, poultry and equine species. The success and benefits of practices such as artificial insemination have stemmed from the fact that they can yield pregnancy rates similar to that of natural conception (Buckley et al., 2003) without the need for a male to be maintained on the farm or breeding facility. Like similar ART procedures in humans, routine ART in farm animals bypasses the natural modes of reproduction. Typically artificial insemination and/or IVF involve procedures such as oestrous manipulation (either to stimulate large numbers of follicles to mature and be ovulated or to synchronise females ahead of insemination/embryo transfer), the collection and preparation of sperm for insemination or storage, IVF and/or the transplantation of embryos into a surrogate. However, as in many other mammalian species, questions over potential long-term effects on the health of the developing fetus and offspring following ART in farm animals (gamete manipulation, IVF, ICSI, embryo culture/transfer), have been raised. In part, these stem from the phenomenon of Large Offspring Syndrome (LOS). Supplementation of the embryo culture media with serum, embryo culture under atmospheric (20%) rather than physiological (~5%) levels of oxygen and supra-physiological hormonal stimulation in the recipient (Ealy et al., 2019) have all been connected with significant changes in patterns of fetal growth, organ development and elevated incidences of postnatal mortality in ruminants. Routine ART practices in humans (IVF, ICSI, embryo culture/transfer) have also been associated with both increased and decreased patterns of fetal growth and altered cardiovascular and metabolic health in the children (Roseboom, 2018). In both farm animals and humans, altered expression of key growth-regulatory imprinted genes has been identified as one mechanism underlying these phenotypic changes (DeAngelis et al., 2018). These observations highlight the sensitivity of the periconception period to sub-optimal environmental conditions, either *in vitro* (e.g. embryo culture media composition) or *in vivo* (parental diet). Such associations underlie the Developmental Origins of Health and Disease (DOHaD) hypothesis (Velazquez et al., 2019). Here, the maturing gametes and preimplantation embryo respond to changes in their immediate environment, resulting in abnormal profiles of epigenetic (DNA methylation, histone modifications, RNA populations) marks being established onto the parental genomes. Post-fertilisation, rates of embryo development, metabolic homeostasis, blastocyst lineage allocation and epigenetic remodelling have all been shown to be altered by sub-optimal environmental conditions (both *in vitro* and *in vivo*), affecting long-term offspring development and health (Fleming et al., 2018) (See Figure 1).

Since the original observations by David Barker, which formed the foundation of the current DOHaD hypothesis (Barker et al., 1989), much of the subsequent epidemiological, clinical and fundamental research has centred on the significance of maternal nutrition and well-being during defined periods of periconception development, gestation and neonatal life. This has been conducted in a range of human, large and small animal models. However, a new focus into the role of the father, the impact his health has on semen quality and how this may affect the long-term health of his offspring has emerged. Previously, the general consensus was that sperm were simply a vehicle carrying the paternal genetic material into the egg, and that the seminal plasma was just a medium to support and transport the sperm. However, we are beginning to understand the epigenetic complexity of the mature sperm, the interaction of the seminal plasma with the maternal uterine environment and the impact that paternal health has on these fundamental reproductive and developmental processes.

While there is a significant drive to use semen from males of high fertility or desirable physical characteristics, a clear understanding of the short-and long-term effects of using sub-optimal semen in a range of farm animals is still lacking. In contrast, studies in humans and mice have allowed for detailed analysis of how poor paternal health at the time of conception modifies the sperm epigenetic landscape, the development of the preimplantation embryo, growth of the fetus and offspring health. In this article, we will focus on the current knowledge base around the connection between male health, semen quality and reproductive fitness in farm animals. First, we will explore how male fertility can be affected by a range of physiological and environmental conditions. In response to these effects, we will then discuss how sperm epigenetic status and seminal plasma composition appear likely mechanisms mediating the paternal programing of offspring health. Finally, we will outline the long-term effects of poor paternal health on offspring well-being.

## Male health and reproductive fitness

In many mammalian species, there are significant differences in fertility between individual males. Studies have shown that in cattle, a general lack of breeding soundness examinations allows for sub-fertile males to be retained within the herd, contributing significantly to failures in herd fertility when compared to bulls used in dairy herds (Flowers, 2013). For many domestic and commercially important farm species, the fertility of an individual male can have a bigger economic impact than that of the females, as breeding males are often used to service multiple females or provide semen for the use in artificial insemination. Furthermore, the use of sub-fertile males can have significant economic and sustainability consequence with increased times to conception, reduced rates of ongoing pregnancy, prolonged seasons of calving and reduced weights of offspring (Thundathil et al., 2016). As such, understanding how environmental factors impact on semen quality and defining the potential post-fertilisation consequences of using semen from males of ‘poor’ reproductive fitness is important for ensuring commercial viability, the effectiveness of conservation programmes and the long-term health and well-being of offspring.

In many large animal species, sperm abnormalities are categorised into ‘compensable’ and ‘uncompensable’. Here, compensable abnormalities can be overcome through the increase in the number of sperm used during artificial insemination. Such abnormalities are associated with an inability for the sperm to reach and fertilise the oocyte, and so are generally considered to be defects in sperm motility. In contrast, uncompensable defects are associated with an inability to maintain pregnancy and the ongoing development of the embryo/fetus. Such defects are likely attributable to chromosomal defects, increased DNA fragmentation and impaired epigenetic status. Interestingly, some studies indicate that routine semen processing procedures such as the cryopreservation or sex-sorting of sperm by flow cytometry may increase the rates of uncompensable defects (Inaba et al., 2016). The cryopreservation of sperm has been of fundamental benefit to the ability to increase genetic diversity within populations and the spread of favourable/superior traits around the world. However, during the cooling and freezing process, sperm can be exposed to several detrimental factors which can negatively affect genomic integrity, membrane composition and metabolic stability (Ugur et al., 2019). During cryopreservation, sperm are susceptible to changes in membrane protein localisation due to lipid phase separation (De Leeuw et al., 1990). Furthermore, sperm shrinkage during cryopreservation may result in elevated levels of reactive oxygen species being released from the mitochondria (McCarthy et al., 2010) which may detrimentally affect DNA integrity. Indeed, sperm freezing and thawing has been linked to increased levels of DNA damage (Lewis and Aitken, 2005), resulting in reduced rates embryo development in both boar (Fraser and Strzezek, 2007) and chickens (Gliozzi et al., 2011). Separate to cryopreservation, the ability to sex-sort sperm to a high degree (over 90%) using flow cytometry has resulted in its use on a commercial level (Garner and Seidel, 2008). However, numerous studies have questioned the impact of the sorting process on sperm and the embryos they generate (Seidel, 2012). Some studies report reduced rates of embryo development with sex-sorted sperm (Inaba et al., 2016; Steele et al., 2020) which may occur in a bull-dependent manner (Barcelo-Fimbres et al., 2011). Additionally, some studies report changes in the number and structure of organelles like mitochondria, rough endoplasmic reticulum and the nuclear envelope in blastocysts derived from sex sorted sperm (Palma et al., 2008).

As in many mammalian species, scrotal temperature is a critical factor in regulating spermatogenesis (Rahman et al., 2018). Elevated scrotal temperature has been associated with increased amounts of abnormal sperm (Skinner and Louw, 1966), increased amounts of sperm cytoplasmic droplets (Koivisto et al., 2009), lipid peroxidation (Balic et al., 2012) and levels of reactive oxygen species (Rhoads et al., 2013). Recent studies have shown that sperm collected from bulls in the spring display higher indices of sperm quality (intact acrosome status, lower reactive oxygen species production, intact mitochondrial membrane potential) then when collected at other times of the year (Sabes-Alsina et al., 2017). Underlying these effects may be seasonal-dependent changes in semen lipid composition. Sperm collected during the summer possessed higher levels of saturated fatty acids and lower levels of polyunsaturated fatty acids and cholesterol than in the winter (Argov-Argaman et al., 2013) associating with decreased proportions of morphologically normal sperm. Additionally, exposure of bulls to a high temperature-humidity index increased rates of sperm death and decreased rates of blastocyst development (Llamas Luceno et al., 2020). Furthermore, elevated scrotal temperature in the bull has also been linked to poor fertilisation capacity of the sperm and perturbed paternal genome DNA demethylation in the zygote (Rahman et al., 2014). Diet can also play a significant role on testicular temperature via its influence on the levels of scrotal fat. Bulls receiving high amounts of dietary energy intake between 6 and 12 or 24 months of age displayed increased amounts of scrotal fat associated with increased scrotal temperature (Kastelic et al., 1997). Daily sperm production and epididymal sperm reserves in these males were significantly reduced when compared to males receiving a control diet (Kastelic et al., 1997).

Separate to the effects in scrotal temperature, nutritional status during calfhood (between 6 and 30 weeks of age) has also been shown to affect the onset of puberty in bulls. Low nutritional intake in calves associated with increased pulsitility of luteinizing hormone and greater testicular development (Barth et al., 2008). Interestingly, these changes were not reversed when males were subsequently given additional feed indicating that these changes, once established, were permanent. On a macro nutrient scale, many studies in ruminants have explored the role of dietary fatty acid supplementation for semen quality. As motility, capacitation and ability to penetrate the oocyte zona pellucida are all influenced by the lipid composition of the sperm plasma membrane, dietary levels of n-3 and n-6 polyunsaturated fatty acids (PUFAs) are important for male reproductive activity (Van Tran et al., 2017). Studies in a range of species including boar (Liu et al., 2015), rabbit (Castellini et al., 2003), chicken (Surai et al., 2000) and bulls (Kaka et al., 2015) have all shown that dietary supplementation with appropriate quantities and ratios of PUFAs can have beneficial effects of sperm lipid composition and male fertility. Interestingly, male age at the time of semen collection/mating appears important for sperm PUFA content. Lower levels of PUFAs (namely Docosahexaenoic acid; DHA) have been reported in semen from older bulls (Argov-Argaman et al., 2013). These observations suggest that as males age, testicular fatty acid metabolism may alter, reducing sperm membrane fluidity and ultimately the capability of the sperm to undergo cryopreservation and/or fertilisation.

In rodents and humans, similar effects on testicular function and sperm quality have been identified in response to a range of dietary and environmental factors such as over- or under-nutrition (McPherson et al., 2014; Watkins et al., 2018), endocrine disruptors (Barakat et al., 2019), stress (Rodgers et al., 2013) and deficiencies in specific nutrients (Lambrot et al., 2013). Here, poor paternal health is associated with defects including altered hormonal profiles, sperm morphological abnormalities and increased DNA fragmentation. Such defects are subsequently linked to reduced fertilisation capacity, lower rates of blastocyst development and reduced rates of ongoing pregnancy and live birth (Colaco & Sakkas, 2018; Ilacqua et al., 2018). While these connections have been widely studied in humans and rodent models, such detailed associations in cattle and large animals are still largely lacking.

Due to the high commercial importance of many breeding males, and with the advances in sequencing technologies, detailed molecular and epigenetic profiling of sperm from males of differing fertility is now being conducted in livestock. Unlike the “biological determinism” paradigm (in which phenotypic characteristics are determined purely by genes), epigenetics provides a molecular mechanism to better interpret the long-term effects the environment has on the emergence of certain phenotypes (Guerrero-Bosagna & Skinner, 2012). Epigenetic mechanisms regulate patterns of gene expression in response to environmental factors and thus act as a link between the environment and an organism’s physiology. Comparison of sperm DNA methylation levels in high fertility and subfertile buffalo/bulls revealed differential methylation at genes for transcription, spermatogenesis, sperm maturation, capacitation and embryo development (Kropp et al., 2017; Verma et al., 2014). In boars, lower levels of DNA methylation at the imprinted *GNAS* complex locus has been identified in sperm from males of lower fertility (Congras et al., 2014). Interestingly, the *GNAS* locus is positioned close to quantitative trait loci for fetal growth and body mass (Thomsen et al., 2004) while gene dosing of *GNAS* is associated with postnatal growth and metabolism (Eaton et al., 2013). Similar imprinting of this locus, and regulation by non-coding transcripts of the several genes it contains, has also been reported in mice and humans (Bastepe, 2007). The role of sperm non-coding RNAs in regulating paternal reproductive fitness is highlighted further by recent studies in mice. Injection of specific tRNA-derived small non-coding RNAs from sperm of high fat diet fed male mice into control zygotes results in impaired glucose metabolism and insulin secretion in the resultant offspring when compared to sperm RNAs from control diet fed males (Chen et al., 2016). Sperm have also been shown to transfer both mRNA molecules (Sharma, 2019) and histones (van der Heijden et al., 2008) to the oocyte, believed to influence early zygotic gene expression. In human and mouse sperm, histones have been identified at key developmental and pluripotency genes such as *Oct4*, *Nanog* and *Sox2* (Hammoud et al., 2011). In cattle, over 6000 transcripts have been identified in sperm with over 60% of them being full length including transcripts for developmentally important transcripts such as *PLCZ1* and *CRISP2* (Card et al., 2013). Analyses of sperm transcript levels between bulls of low or high fertility have shown deficits in genes related to gene transcriptional and translational regulation (Feugang et al., 2010). In addition to sperm transcript levels, studies have identified levels of sperm histone methylation (H3K27me3) and acetylation (H3K27ac) as markers of male fertility in Holstein bulls (Kutchy et al., 2018). Furthermore, sperm from bulls with low fertility have been shown to display less DNA condensation, perturbed protamine exchange and increased DNA damage relative to sperm from higher fertility bulls (de Oliveira et al., 2013; Dogan et al., 2015).

As with other aspects of semen quality, the age at which semen is collected from a bull may also affect the epigenetic status of the sperm. DNA methylation profiles have been shown to differ in sperm collected from early pubertal, late pubertal, and pubertal bulls (Lambert et al., 2018). Separately, semen samples collected from bulls younger than 1 year of age have been shown to have lower sperm motility profiles than bulls older than 1 year (Murphy et al., 2018). However, post-thaw viability of sperm from the young bulls was comparable to that of the older bulls. Despite multiple studies demonstrating altered sperm epigenetic status in association with male age or fertility, the potential long-term impact(s) of using these sperm is still to be defined.

## The importance of seminal plasma

While the impact of sperm epigenetic status on embryo development and offspring health has received detailed investigation, the significance of the seminal plasma has been overlooked. Typically, seminal plasma has been viewed as medium for supporting and transporting the sperm through the female reproductive tract. However, studies in mice have shown that following insemination, significant influxes of leucocytes are observed within the female reproductive tract for up to 72 hours accompanied by significant increases in the expression of numerous inflammatory mediators (Robertson et al., 1996). Specifically, studies have shown that seminal plasma Tgfb1 and granulocyte colony macrophage stimulating factor (Csf2) are significant mediators of post-fertilisation uterine responses (Robertson et al., 2018). In humans, similar changes in cervical immune responses to the presence of seminal plasma have been identified, which are absent following intercourse with the use of condoms (Sharkey et al., 2012). Interestingly, as in humans, embryo implantation and fetal development can occur in the absence of seminal plasma in cattle (Faulkner et al., 1968). However, some studies indicate TGFβ infusion of at the time of insemination can improve pregnancy rates in cows, especially in low fertility herds (Odhiambo et al., 2009). In ruminants, proteins which can bind to sperm and can both stimulate and inhibit sperm function have been identified in seminal plasma (Maxwell et al., 2007). Additionally, aspects of sperm quality including motility and chromatin integrity have been shown to be altered in response to differential compositions of bovine seminal plasma (Garner et al., 2001; Maxwell et al., 1996), while analysis of seminal plasma composition between bulls of high fertility and bulls of low fertility have identified differences in the profiles of specific proteins (D’Amours et al., 2010; Killian et al., 1993). Seminal plasma in the pig has similarly been shown to improve sperm survival and motility (Chutia et al., 2014). Here, unwashed boar sperm held at 15^o^ C for 24 to 72 hours in commercial GEPS extender showed significantly higher motility, survival and integrity of the acrosome prior to preservation when compared to washed sperm held in GEPS alone (Chutia et al., 2014). Interestingly, uterine seminal plasma deposition in the pig has been shown to not only influence uterine prostaglandin synthesis gene (*PTGS2*) expression but also the expression of multiple maturation promotion factors within the oocyte, cumulus and granulosa cells within the ovary (Waberski et al., 2018). Furthermore, uterine inflammatory responses persist for up to 8 days post seminal plasma infusion (O’Leary et al., 2004) and significant increases in the number of viable embryos being collected post insemination have been reported in the pig (O’Leary et al., 2004). In equine species, including the horse and donkey, seminal plasma has been shown to have a role in uterine priming by directing the expression of pro- and anti-inflammatory cytokines (e.g. *IL-8, IL-1B*, *TNF* and *COX2*) in uterine endometrial tissue (Fedorka et al., 2017; Vilés et al., 2013). There is also evidence that, in horses, sperm have the ability to initiate an inflammatory-response in the uterus by recruitment of neutrophils (Kotilainen et al., 1994). In the absence of seminal plasma however, endometrial neutrophils have been found to phagocytose stallion spermatozoa, yet in the presence of seminal plasma viable sperm are protected (Asbury & Hansen, 1987; Troedsson et al., 2001). Furthermore, conception rates in mares are associated with seminal plasma availability, with a conception rate of just 5% reported in cases of artificial insemination without seminal plasma, verses 77% when conducted in the presence of seminal plasma (Alghamdi et al., 2004).

Changes in seminal plasma composition have also been linked with fertility in men. When compared to seminal plasma from fertile men, lower levels of prostaglandin-D synthase (PGDS) have been identified in men with azoospermia (Heshmat et al., 2008). Conversely, azoospermic men display elevated levels of seminal prolactin-inducible protein (PIP), galectin-3-binding protein (LGALS3BP) and prostatic acid phosphatase (PAP) (Davalieva et al., 2012) when compared to fertile men. Additionally, proteins such as human cationic antimicrobial protein (hCAP18), lactoferrin and Semenogelin I and II have been identified as being important for fertility in men (Milardi et al., 2012).

In addition to the protein composition of the seminal plasma, there is now a large interest in the role exosomes may have in regulating male fertility. Epididymal exosomes (epididymosomes) contain a range of proteins, microRNAs, tRNA-derived small RNAs (tsRNAs) and fluid and are able to interact with the mature sperm. Analysis of epididymosomes from mice have identified over 350 miRNAs with approximately 60% of them being detectable in the sperm (Reilly et al., 2016). Furthermore, male mice fed a high fat diet display altered miRNA profiles in their sperm, potentially originating from the epididymosomes, which have then been shown to affect offspring development and health (Grandjean et al., 2015). Similarly in humans, seminal plasma also contains a range of tsRNAs which may act to regulate immune responses within the female reproductive tract (Vojtech et al., 2014).

## Paternal effects on offspring health

The continued development and use of assisted reproduction technologies amongst farm animals has vastly improved the economic burden of reproductive inefficiency and the productivity of livestock. However, whilst these technologies have enhanced rates of conception and the number of offspring produced, considerations must still be given to the health of the offspring generated. A multitude of paternal factors that can influence offspring health have now been identified, including age, environmental exposures and nutritional status (Fullston et al., 2017). In cattle, a recent study identified 25 paternal candidate genes and differential profiles of sperm DNA methylation that associated with maternal gestation length (Fang et al., 2019). Such effects could conceivably affect fetal growth and weight at birth, factors known to influence adult risk for cardio-metabolic diseases. As discussed earlier, there is evidence that the phenomenon of LOS in cattle is influenced by epigenetic changes that result in alterations of imprinted genes, including *H19/IGF2* regions and the paternally expressed long non-coding RNA *KCNQ1OT1 *(Hori et al., 2010; Robbins et al., 2012). It would be of interest to study whether using sperm from low versus high fertility males (known to display differential epigenetic status) affects the incidences and severity of LOS in their offspring. Furthermore, one of the few porcine studies investigating the impact of paternal diet observed that supplementing paternal diet with methyl donors (dietary factors used for the methylation of DNA and histones) lowered fat percentage in F2 offspring coinciding with significant differences in liver DNA methylation, suggesting paternal diet epigenetically programed offspring fat metabolism pathways over multiple generations (Braunschweig et al., 2012). In contrast, methyl donor supplementation in male mice results in significant sperm DNA hypermethylation of genes linked to olfaction and impairments in adult offspring cognitive performance (Ryan et al., 2018).

In mice, transgenerational effects of paternal diet have also been demonstrated. Here, suboptimal paternal low protein diet impaired F1 and F2 mouse offspring cardiovascular function and the rennin-angiotensin system (RAS) activity through both sperm and seminal plasma mediated pathways (Morgan et al., 2020). Underlying these effects on offspring health were significant changes in sperm DNA methylation, testicular expression of central epigenetic regulators and maternal preimplantation uterine immunological mediated responses (Watkins et al., 2018). Other nutritional impairments in the father, such as obesity, have been linked to metabolic dysregulation and sub-fertility in offspring and grand-offspring in mice, implicating poor paternal nutritional status with the development of 2 subsequent generations (Fullston et al., 2015). Paternal obesity in mice was found to result in growth-restricted fetuses with abnormal limb development and placental insufficiency (Binder et al., 2015; Binder et al., 2012). Fetal growth restriction, where a fetus fails to reach its genetic growth potential, is associated with poorer neonatal survival, as well as impaired cardiovascular and metabolic functions in adulthood (Barker et al., 2002; Felicioni et al., 2020; Wallace et al., 2020). Paternal obesity has also been found to impair fertility in female offspring (Fullston et al., 2015), thus impacting the production of future generations. Furthermore, absence of the seminal plasma at the time of conception in mice has been shown to impair embryo development and cell number as well as the adiposity and metabolic health of adult offspring (Bromfield et al., 2014).

## Discussion

As with human reproduction, ART in farm animals has revolutionised fertility management on a global scale. While the application of such technologies has enhanced our capacity to treat human infertility and make agricultural practices more efficient, we must be mindful of the potential implications for offspring health. Both human and animal ART are associated with significant implications for the resultant offspring. Here, both increases and decreases in fetal growth and weight at birth have been reported, resulting in complications with postnatal offspring wellbeing and development. Underlying such offspring effects are factors including a failure to recapitulate the natural maternal *in vivo* environment, manipulation of the gametes and the use of sub-quality gametes. The latter can be a direct consequence of the health of the parents at the time of conception. Poor nutritional status in a range of animal models has been shown to negatively impact gamete quality, fertility and post-fertilisation development. While a significant focus has been to define and improve maternal reproductive fitness, sperm quality and an understanding of the potential long-term consequences of poor paternal health have remained overlooked. From a large animal model perspective, our understanding of the long-term paternal effects is still very limited. The majority of studies so far have been conducted in rodents and humans. While these have provided detailed mechanistic insight into the potential molecular and epigenetic mechanisms through which paternal health links to sperm quality and offspring well-being, data from mice and humans cannot always be extrapolated directly to other mammalian species. Furthermore, a greater understanding of the role of the whole semen, and not just the sperm in isolation, is needed to understand male reproductive fitness effects. In both agricultural and human ART, procedures are routinely conducted in a predominantly seminal plasma free environment followed by the transfer of an embryo into a non seminal-primed uterus. Data from rodent (Bromfield et al., 2014; Morgan et al., 2020; Watkins et al., 2018) and human (Coulam & Stern, 1995; Marconi et al., 1989; Tremellen et al., 2000) studies both show potential reproductive benefits of uterine exposure to seminal plasma around the time of conception and embryo implantation. Therefore, it is imperative that we develop a better understanding of the impacts male health has not only for the benefits of his own health, but also for the health of the mother and ultimately, the health of his offspring.
